# Improved evidence-based genome-scale metabolic models for maize leaf, embryo, and endosperm

**DOI:** 10.3389/fpls.2015.00142

**Published:** 2015-03-10

**Authors:** Samuel M. D. Seaver, Louis M. T. Bradbury, Océane Frelin, Raphy Zarecki, Eytan Ruppin, Andrew D. Hanson, Christopher S. Henry

**Affiliations:** ^1^Mathematics and Computer Science Division, Argonne National LaboratoryArgonne, IL, USA; ^2^Computation Institute, The University of ChicagoChicago, IL, USA; ^3^Horticultural Sciences Department, University of FloridaGainesville, FL, USA; ^4^Department of Biology, York College, City University of New YorkNew York, NY, USA; ^5^Sackler Faculty of Medicine, Tel Aviv UniversityTel Aviv, Israel

**Keywords:** systems biology, plant metabolism, transcriptomics, metabolic networks, flux balance analysis, *Zea mays*

## Abstract

There is a growing demand for genome-scale metabolic reconstructions for plants, fueled by the need to understand the metabolic basis of crop yield and by progress in genome and transcriptome sequencing. Methods are also required to enable the interpretation of plant transcriptome data to study how cellular metabolic activity varies under different growth conditions or even within different organs, tissues, and developmental stages. Such methods depend extensively on the accuracy with which genes have been mapped to the biochemical reactions in the plant metabolic pathways. Errors in these mappings lead to metabolic reconstructions with an inflated number of reactions and possible generation of unreliable metabolic phenotype predictions. Here we introduce a new evidence-based genome-scale metabolic reconstruction of maize, with significant improvements in the quality of the gene-reaction associations included within our model. We also present a new approach for applying our model to predict active metabolic genes based on transcriptome data. This method includes a minimal set of reactions associated with low expression genes to enable activity of a maximum number of reactions associated with high expression genes. We apply this method to construct an organ-specific model for the maize leaf, and tissue specific models for maize embryo and endosperm cells. We validate our models using fluxomics data for the endosperm and embryo, demonstrating an improved capacity of our models to fit the available fluxomics data. All models are publicly available via the DOE Systems Biology Knowledgebase and PlantSEED, and our new method is generally applicable for analysis transcript profiles from any plant, paving the way for further *in silico* studies with a wide variety of plant genomes.

## Introduction

The ability of a plant to grow and survive is linked to its metabolic network (Stitt et al., 2010), which indicates that a capacity to predict and understand plant metabolism will improve our understanding of plant response to changing environments and genetic perturbations (Mo et al., 2009; Chang et al., 2011; Saha et al., 2011). Furthermore, the yield of a wide range of plant products is crucial to human society, particularly when inputs such as water are limited (Skirycz and Inze, 2010). Many classical biochemical and genetic experiments involve the elucidation of biological functions for individual gene products. However, many external and internal perturbations lead to systemic responses, and a systems-level understanding of plant metabolism is required to fully explain these system responses.

To build this systems-level understanding, several genome-scale metabolic reconstructions have recently been published for plant species (Poolman et al., 2009; de Oliveira Dal'molin et al., 2010a,b; Saha et al., 2011; Poolman et al., 2013). Each reconstruction consists of all reactions known to be catalyzed by one or more of the gene products in the plant genome. The methods employed to study these metabolic models, such as flux balance analysis (FBA), consider all reactions in the model when attempting to predict a biological phenotype, such as plant growth. Metabolic reconstructions are built from many data sources, notably public databases and individual publications. Reconstructions are validated by comparing the activity of well-characterized pathways *in silico* with biochemical evidence in the literature. Poolman et al. (2009) built the first genome-scale plant metabolic reconstruction, which could respire on heterotrophic media *in silico* and produce biomass components in proportions that matched *in vivo* observations. de Oliveira Dal'molin et al. (2010a) investigated autotrophic biosynthesis of plant biomass, showing that the model correctly predicted the reactions used for both photosynthesis and photorespiration. de Oliveira Dal'Molin et al. also developed a metabolic reconstruction of a C4 plant (de Oliveira Dal'molin et al., 2010b) containing plastidial reactions for photosynthesis. This reconstruction was shown to be capable of performing three known subtypes of C4 photosynthesis. In other work, Saha et al. (2011) show that genetic perturbations in the phenylpropanoid biosynthesis pathway could be simulated *in silico*, producing an impact on cell wall composition that compared favorably with experimental data from known maize mutants.

The validation approaches described above are based on a few well-known biochemical pathways, and involve large genome-scale metabolic reconstructions, built to provide a systems-level understanding of how a metabolic network behaves under certain conditions. For example, Schwender and Hay (2012) investigated how a metabolic reconstruction exhibited variation in reaction activity in response to variation in the biosynthetic demands of oil and protein as storage products in the plant embryo and were able to identify the utilization of a pathway within the network of reactions that was not yet characterized in the literature. Similarly, Töpfer et al. (2013) explored the means with which a set of pathways in a metabolic reconstruction responded to various conditions of light and temperature, showing, in one case, the preference for methylerythritol 4-phosphate pathway over the mevalonate pathway in isoprenoid biosynthesis, and also generating a new hypothesis for the role of homocysteine–cysteine conversion.

Genome-scale metabolic reconstructions are generated based on the annotation of all gene products in the full genome, and, thus, they include every reaction that can be catalyzed by the plant. However, a multi-cellular organism will activate different subsets of their genes in different organs, tissues, developmental stages, and environmental conditions. To be accurate, genome-scale metabolic reconstructions must represent the reduced metabolism that truly exists in cells of a specific type and in a specific condition. Most reconstructions mentioned previously were either intended to represent a leaf cell or the primary metabolism of a generic plant cell. Other metabolic reconstructions have been built to target specific tissues and organs, such as the seeds of barley (*Hordeum vulgare*; Grafahrend-Belau et al., 2009), and the embryos of oilseed rape (*Brassica napus*; Hay and Schwender, 2011a,b; Pilalis et al., 2011). Grafahrend-Belau et al. followed up their study of barley seeds by building manually curated metabolic reconstructions of barley stem and leaf, and integrating the three reconstructions into a single model (Grafahrend-Belau et al., 2013). Recently, several new approaches have emerged to integrate large-scale data (Baerenfaller et al., 2008) in an automated manner to either generate new condition-specific models (Mintz-Oron et al., 2012), or to constrain the behavior of individual reactions in a full genome-scale model to better reflect the behavior of specific organs or tissues (Töpfer et al., 2013).

The ongoing explosion in plant transcriptome sequencing, driven by advances in next-generation sequencing (NGS) and by the relative ease of sequencing a collection of cDNAs as opposed to predicting gene models in plant chromosomes (Ozsolak and Milos, 2011), means that many transcript profiles are now publicly available, and individual laboratories can afford to generate new transcript profiles for individual experiments. Indeed, Töpfer et al. used their own transcript profiles, which they generated from Arabidopsis rosettes (Töpfer et al., 2013). Several computational methods have been developed that are able to integrate transcript profiles with a metabolic reconstruction to produce improved predictions of reaction utilization and flux.

Töpfer et al. used E-flux (Colijn et al., 2009), which fits flux predictions based on gene expression data, but does not attempt to reduce a full genome model to a tissue or organ specific version. The Töpfer et al. work was focused on several primary and secondary metabolic pathways that are known to be active with the rosettes of Arabidopsis. Mintz-Oron et al. used the iMAT approach (Jerby et al., 2010; Zur et al., 2010), which generates aggregate models based on random sampling of fluxes to fit gene expression data. While this approach provides a more comprehensive account of the metabolic network, the extensive sampling can be cumbersome. An updated method eliminates the need for random sampling and thereby runs faster (Wang et al., 2012). This method searches for an optimal solution by iteratively activating each reaction whose associated genes have high expression, which means that the method still performed many optimizations. We have developed a new approach that requires far fewer optimization steps, allowing for transcriptome-based metabolic reconstructions to be formed from transcript profiles at a greater speed and with less complexity. We note here the introduction of the term transcriptome-based to reflect this class of model, which is based on fitting a genome-scale model to a select subset of gene expression data. The term tissue-specific is often used for models of this type. However, expression data often does not capture the entire behavior or a tissue, nor does a single tissue necessarily reflect a single biological behavior (e.g., leaf tissue consists of several sub-cell types).

We demonstrate our approach for reconstruction of transcript-specific models with a new genome-scale metabolic reconstruction of maize. Our new genome-scale maize model includes three important enhancements over previously published models: (i) an expanded and improved biomass composition; (ii) improved gene-protein-reaction associations where low confidence gene-reaction mappings based on poor evidence or purely computational predictions have been removed; and (iii) improved compartmentalization of reactions to subcellular organelles based on a combination of literature evidence, curation, and gapfilling algorithms. The improved gene-reaction associations in our new model were critical to our use of maize transcript profiles (Davidson et al., 2011) to produce new transcriptome-based models of the leaf, embryo, and endosperm in maize. We applied our novel model reconstruction method to maximize the activity of reactions associated with high expression genes while removing as many reactions associated with low expression genes as possible. We also adjusted the biomass composition of our embryo and endosperm models to better fit the actual composition data for these tissues by curating data for individual components from a variety of literature sources. To test the accuracy of our models, we explored how well they replicate the flux profiles measured for central carbon metabolism in embryo and endosperm tissues (Alonso et al., 2010, 2011). This analysis demonstrates that our models have an improved fit between the fluxes generated *in silico* and the fluxes measured *in vivo*. All models produced from this work are available for download from the DOE Systems Biology Knowledgebase (http://kbase.us) and the PlantSEED resource (Seaver et al., 2014).

## Materials and methods

### Biochemistry

We used the plant biochemistry database built for the PlantSEED project (Seaver et al., 2014). This database is notably built on KEGG (Kanehisa and Goto, 2000; Kanehisa et al., 2012) and MetaCyc (Caspi et al., 2012), which had been integrated using InChI (Heller et al., 2013) strings generated from mol files provided by both databases. The integration includes several plant biochemistry databases such as the BioCyc databases for *Arabidopsis thaliana* (*Arabidopsis*; AraCyc v11.5 Mueller et al., 2003; Zhang et al., 2010), and maize (MaizeCyc v2.2.2 Monaco et al., 2013 and CornCyc v4.0 Zhang et al., 2010), and several published metabolic models for *A. thaliana* (de Oliveira Dal'molin et al., 2010a,b, 2011; Saha et al., 2011; Mintz-Oron et al., 2012) and maize (de Oliveira Dal'molin et al., 2010b; Saha et al., 2011). The metabolic reconstructions we built depend on this integration, and the reactions for the respective *Arabidopsis* and maize metabolic reconstructions are thus drawn from this database.

### Compartments

An important aspect of plant metabolic models is the compartmentalization of reactions into plastids, mitochondria, and other organelles. To accurately capture this compartmentalization, we downloaded localization data for proteins from PPDB (Sun et al., 2009), SUBA (Tanz et al., 2013), AraCyc, MaizeCyc, and CornCyc. We systematically avoided any protein localizations generated solely via computational predictions. From PPDB, we only used data that the PPDB team had curated. From SUBA, we only used data from GFP experiments, which are more reliable than the data from mass spectrometry experiments. Finally, from AraCyc, MaizeCyc, and CornCyc, many reactions are localized according to biochemical support such as the histidine pathway in plastids (Ingle, 2011). Even if the genes associated with these pathways do not have localization data, we considered them to be localized if there was experimental evidence for the gene-reaction associations. Much of the localization data could only be applied directly to either of the two different species, and therefore we propagated the associations between *Arabidopsis* and maize by using the same conservative approach we applied to EnsemblCompara protein families in the PlantSEED project (Vilella et al., 2009; Kersey et al., 2014; Seaver et al., 2014).

### Model pathway-gapfilling

A new gapfilling algorithm was applied during the reconstruction of all our plant genome-scale models. This algorithm provides a means of identifying the minimal set of reactions that must be made reversible or added to the model in order to activate as many gene-associated reactions in the model as possible. The constraints of the optimization problem resemble the constraints for existing classical gapfilling approaches (Satish Kumar et al., 2007; Kumar and Maranas, 2009).

(1) $$N_{super}•v=0$$

(2) $$0\leq v_{i}\leq 100z_{i}\;i=1,\ldots ,r_{gapfill}$$

(3) $$z_{for,i}+z_{rev,i}\leq 1\text{ ​​ }i=1,\ldots ,r_{gapfill}$$

(4) $$-100\leq v_{ex,i}\leq 100\gamma_{i}\;i=1,\ldots ,m_{transported}$$

Equation (1) represents the mass balance constraints, where *N*_*super*_ is the matrix of stoichiometric coefficients through all reactions in our model plus all candidate reactions added from our biochemistry database, while ***v*** is the vector of fluxes through all model and database reactions represented in the *N*_*super*_ matrix. In these and all other constraints, reversible reactions have been decomposed into separate forward and backward component reactions to ensure that all fluxes are always positive. Equation (2) sets the bounds on the flux through reaction *i*, where *v*_*i*_ is the flux and *z*_*i*_ is a binary use variable equal to zero when the flux is zero and equal to one otherwise. Equation (3) ensures that the forward and backward components of the same reaction may not both be active at the same time; in our formulation, this constraint is the sole reason for using binary variables. Equation (4) establishes the growth conditions for the gapfilling analysis; metabolites present in the growth media (e.g., heterotrophic media or autotrophic media) have a γ_*i*_ of 1 in Equation (4). Otherwise γ_*i*_ is zero.

In addition to these standard constraints, we applied a new constraint that introduces a slack flux for all reactions found in the original un-gapfilled model:

(5) $$v_{for,i}+v_{rev,i}+\delta_{i}\geq 0.01\;i=1,\ldots ,r_{model}$$

Equation (5) states that the sum of the net flux through reaction *i* (*v*_*for*, *i*_ + *v*_*rev*, *i*_) and the slack flux for reaction *i* (δ_*i*_) must be greater than or equal to 0.01. As a result of this constraint, a reaction can only have a net flux of zero if the corresponding slack flux is 0.01. Thus, the slack flux is a variable used to identify reactions that carry no flux in the model. We utilize this new slack flux for this purpose in the objective function for our gapfilling.

#### Objective:

(6) $$\text{Minimize}\sum_{i\;=\;1}^{r_{annotated}}a\left(\gamma_{activate,i}\delta_{i}\right)+\sum_{i\;=\;1}^{r_{gapfilling}}\left(\gamma_{gapfill,i}v_{i}\right)$$

This new objective function minimizes the sum of the slack fluxes associated with the reactions included in our original model while simultaneously minimizing the flux through all gapfilled reactions added to the model from our database. The purpose of this objective function is to maximize the number of gene-associated reactions that carry flux while minimizing the number of gapfilled reactions added to the model. This effectively gives precedence to the gene-associated reactions in our model. The activation coefficient, γ_*activate*, *i*_, dictates the cost of leaving a gene-associated reaction inactive, while the gapfilling coefficient, γ_*gapfill*, *i*_, dictates the cost of adding a gapfilled reaction to the model. In our gapfilling studies, we set γ_*activate*, *i*_ equal to one for all gene-associated reactions, while we computed γ_*gapfill*, *i*_ as described in our previous work (Henry et al., 2009, 2010).

We also used a scaling factor *a* in our objective function, which scales the cost of leaving some model reactions inactive against the cost of adding new reactions to the model from the database. We explored values for *a* ranging from 0.01 to 0.25, but we found only a small effect on the solutions produced. Generally, an *a* of 0.1 generated the most well-balanced gapfilling solutions.

In this gapfilling formulation, we utilize continuous linear flux variables in our objective function rather than the more typical binary variables (e.g., *z*_*for*, *i*_ and *z*_*rev*, *i*_) (Kumar et al., 2007). This adjustment reduced the compute time required to obtain a globally optimal solution by over 90% while having no appreciable impact on solutions obtained. This use of linear variables has been previously proposed in other published gapfilling algorithms, with detailed sensitivity analyses performed and similar results obtained (Latendresse, 2014). Thus, we do not repeat the sensitivity analysis here.

### Transcriptome-based pathway-gapfilling

Our method for producing transcriptome-based models builds on the pathway-gapfilling approach (see previous used during the reconstruction of our models. Our pathway-gapfilling approach attempts to maximize the number of number of active *gene-associated* reactions. This approach further refines the model toward a specific transcriptome by maximizing the activity of reactions associated with highly expressed genes while minimizing active reactions associated with minimally expressed genes. This formulation includes flexibility permitting high-expression reactions to remain “off” if activating them requires the function of too many low expression reactions, and vice versa.

The first step of this algorithm is to categorize every reaction in the model as either high expression or low expression. This is done by assigning an expression score, *E*_*exp*, *i*_, to every gene-associated reaction *i* as follows:

(7) $$E_{exp,i}=Max(C_{exp,i,j})\;i=1,\ldots ,r\text{​​ }j=1,\ldots ,c_{i}$$

(8) $$C_{exp,j}=Min(P_{exp,j,k})\;j=1,\ldots ,c_{i}\text{​​​​ }k=1,\ldots ,p_{j}$$

(9) $$P_{exp,k}=Max(G_{exp,k,l})\text{ ​​}k=1,\ldots ,p_{j}\text{​​​​ }l=1,\ldots ,g_{k}$$

In Equations (7)–(9), the reaction expression score, *E*_*exp*, *i*_, is equal to the maximum of the complex expression scores, *C*_*exp*, *i*, *j*_ for all *c*_*i*_ protein complexes catalyzing reaction *i*; the complex expression scores are equal to the minimum of the protein expression scores, *P*_*exp*, *j*, *k*_, for all *p*_*j*_ protein subunits of each complex *j*; and the protein expression scores, are equal to the maximum of all gene expression scores, *G*_*exp*, *k*, *l*_, associated with the *g*_*k*_ genes encoding each protein subunit. The gene expression score is equal to the normalized expression value of gene in the transcriptome being used as the basis to construct the model. In our analysis, the expression value of each gene was normalized by the median expression value for the same gene across all 37 conditions included in our data set, which included data from numerous organs, tissues, and growth conditions.

Reactions with an expression score falling below 0.2 were categorized as being “low expression.” Biologically, a score of 0.2 means that the critical genes associated with the reaction are expressed at 20% of their average expression across all 37 conditions included in our transcriptomics data. This represents a conservative calling of “low expression” genes. We then applied the gapfilling algorithm as described in Equations (1)–(6) with two modifications: (i) the mass-balance constraints encoded by Equation (1) only included the stoichiometry of the reactions in the gapfilled full genome model (stoichiometry was not expanded to include the entire biochemistry database as done in full gapfilling); and (ii) the objective function was altered to maximize the high expression reaction activity while minimizing flux through low-expression reactions (Equation 10).

#### Objective:

(10) $$Minimize\sum_{i\;=\;1}^{r_{high}}a\left(E_{exp\text{-}high,i}\delta_{high,i}\right)+\sum_{i\;=\;1}^{r_{low}}a\left(E_{exp\text{-}low,i}v_{low,i}\right)$$

Similar to our gapfilling formulation, this objective function minimizes the flux through the low expression reactions while also minimizing the slack fluxes associated with all high expression reactions. This maximizes the number of high expression reactions with a non-zero flux while setting the flux through as many low expression reactions as possible to zero. Again, we use a scaling factor *a* in our objective function, which scales the cost of leaving some high expression reactions inactive against the cost of activating some low expression reactions. We explored values for *a* ranging from 0.01 to 0.25, with only minimal effect on the solutions produced. We found an *a* of 0.1 generated the most well-balanced solutions.

### Comparison with estimated fluxomics data for embryo and endosperm

In order to calculate how well the metabolic model can match experimentally measured flux data for a list of specific reactions, we applied a QP where we minimized the distance between the predicted fluxes and the experimentally measured fluxes. The QP utilized the standard FBA constraints:

(11) $$N_{model}•v=0$$

(12) $$v_{min,i}\leq v_{i}\leq 1000\;i=1,\ldots ,r_{model}$$

(13) $$-50\leq v_{ex,i}\leq 50\gamma_{i}\;i=1,\ldots ,m_{transported}$$

Equation (11) represents our mass balance constraints, where *N*_*model*_ is the stoichiometry matrix for all model reactions and ***v*** is the vector of fluxes through all model reactions. Unlike our gapfilling formulation, in this study, reversible reactions were not decomposed. Equation (12) represents the bounds on the flux through each reaction, with the lower bound *v*_*min*, *i*_ being zero if a reaction is irreversible and −1000 is a reaction is reversible. As in our gapfilling formulation, Equation (13) sets the bounds of uptake of nutrients from the environment.

In the quadratic objective function of our QP, we minimize the deviation of our predicted fluxes (*v_*i*_*) from the experimentally measured fluxes (*v*_*exp*, *i*_):

(14) $$Minimize\sum_{i\;=\;1}^{r_{measured}}{\left(v_{exp,i}-v_{i}\right)}^{2}$$

This approach is similar to that adopted by Lee et al. (2012), but by using QP, we find a single solution and avoid the iterative approach they describe. The calculations were done when the model was grown on heterotrophic media. After the minimal distance between experimental and model predicted fluxes was found via the QP problem as described above, we performed a Spearman correlation between the experimental flux values and the actual predicted flux values found by the solution when the model reached the minimal distance. The results in the form of the Spearman value and the *p*-value of the Spearman correlation are shown in **Table 2**.

## Results

### A high-quality evidence-based genome-scale metabolic reconstruction of maize

In order to generate a metabolic reconstruction based on available evidence, as described in the Materials and Methods Section, we started by building a full genome-scale metabolic reconstruction that integrated every reaction and gene-reaction association from all available resources. We then refined this model by removing the reactions and gene-reaction associations that did not have available support such as literature citation, human curation, or notation of presence in a specific compartment. We call this refined model an *Evidence-Based Model*. Here we described the process applied to complete this model refinement.

#### Initial Reconstruction of Full Genome-Scale Metabolic Models

We built our initial genome-scale metabolic reconstructions for *Arabidopsis* and maize using all reactions and genes obtained from all available resources. The resources included KEGG, the respective BioCyc databases, and the respective published metabolic models for *Arabidopsis* and maize (de Oliveira Dal'molin et al., 2010a,b; Saha et al., 2011). The two initial reconstructions are named “Full” and were composed of 6399 total reactions for *Arabidopsis* and 6458 for maize (Table 1).

**Table 1:** **A list of metabolic models generated in our work and their statistics**.

**Species**	**Type/Organ/Tissue**	**Reactions**	**Compounds**	**Gene-reaction associations**	**Gapfilled reactions**
*Arabidopsis thaliana*	Full	6399	6236	16,577	1073
*Arabidopsis thaliana*	Evidenced	2801	2864	4262	697
*Zea mays*	Full	6458	6250	35,226	979
*Zea mays*	Evidenced	2629	2634	5540	667
*Zea mays*	Evidenced/Leaf	2322	2635	4656	925
*Zea mays*	Evidenced/Embryo	2304	2636	4680	885
*Zea mays*	Evidenced/Endosperm	2280	2636	4602	920

Although we used multiple sources, we note that every published metabolic model available was in turn derived from KEGG and the respective BioCyc database. These databases are dynamic and improved over time, and, as a consequence, the published models are considered outdated. We therefore did not fully integrate the published metabolic models with two important exceptions: transport reactions and organellar reactions. These two sets of reactions, with the exception of those present in the model generated by Mintz-Oron et al. (2012), were manually reviewed in order to ensure that intra-organellar metabolic networks were active. We therefore ensure that these reactions are included.

The most telling statistic in comparing the Full metabolic reconstructions for both species is that maize has many more gene-reaction associations. This is partly because maize has undergone a recent whole-genome duplication event (Schnable et al., 2009), thus creating many paralogs, and partly because, for the MaizeCyc and CornCyc databases, many gene-reaction associations were predicted, and thereby included many similar homologs.

**Figure 1:**
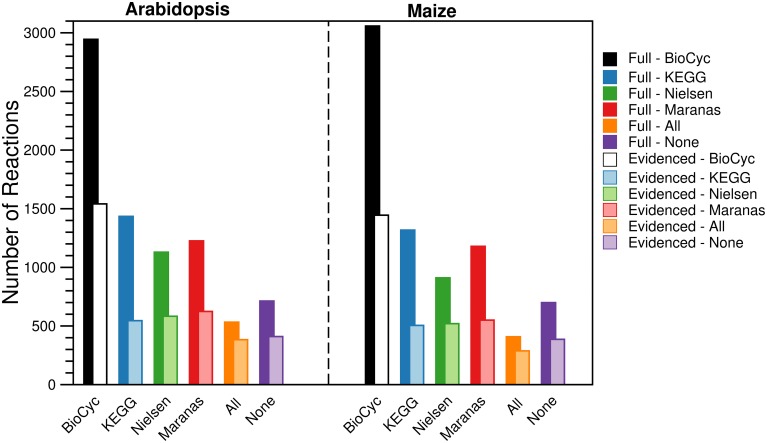
**Number of reactions in the Full and Evidenced metabolic reconstructions for *Arabidopsis* and maize**. The bars represent the number of reactions shared with each of the four primary biochemical sources used to build the Full metabolic reconstruction. Reactions are counted multiple times if they are present in multiple compartments. The “All” category corresponds with reactions that are shared between all four sources, and the “None” category corresponds with reactions present in compartments that are not otherwise found in the primary sources due to protein localization evidence. The dominant source of reactions was the BioCyc databases, ~50% more reactions originated from AraCyc and MaizeCyc/CornCyc than from KEGG. In addition, the dominant source of evidence for gene-reaction evidence came from AraCyc, and as a result, far fewer reactions are shared between the Evidenced metabolic reconstructions and the published counterparts, which were originally derived from KEGG.

**Figure 2:**
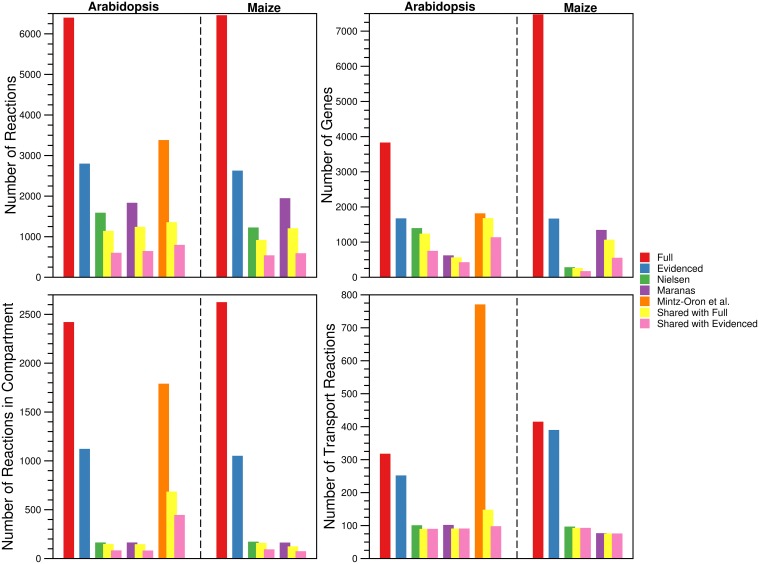
**Comparison of the number of reactions, genes, compartmentalized reactions, and transport reactions found in the Full and Evidenced metabolic reconstructions for Arabidopsis and Maize and the published metabolic models**. For each of the published metabolic models, we also show the number of reactions, genes, compartmentalized reactions, and transport reactions that are shared with both the Full and Evidenced metabolic reconstructions. The number of reactions and genes in the Full metabolic reconstructions dwarf the numbers in the other models. The model generated by Mintz-Oron et al. was the first plant metabolic model to be published for which integration from more than biochemical source was performed, and as such, it has more reactions than the other published models. However, AraCyc has since gone through several expansions, which explains why so many more reactions are in the Full metabolic reconstructions (Figure 1). The high number of genes in the Full maize model is indicative of the number of paralogs for which computational predictions are made by multiple sources. Only 40% of the genes in iRS1563 are found in the Evidenced maize metabolic reconstruction. The Evidenced metabolic reconstructions contain over 1000 reactions that are found in other compartments (notably in the plastid, see **Figure 4**), which is approximately 10 times more than the number of compartmentalized reactions found in the models from the Nielsen and Maranas labs. The process of creating the metabolic model of Mintz-Oron et al. predicted many more compartmentalized and transport reactions than those found in the Evidenced metabolic reconstruction for Arabidopsis, but only 25% of the compartmentalized reactions and 13% of the transport reactions are found in the Evidenced metabolic reconstruction.

For each metabolic reconstruction, we showed the number of reactions that came from each source in Figure 1. In the Evidenced models, most of the reactions originated from BioCyc databases because KEGG provides comparatively little literature evidence for gene-reaction associations. In contrast, there is significant overlap between the KEGG database and the metabolic models published by the Nielsen/Maranas groups. This is because those metabolic models were generated from KEGG alone. We also highlight the variation in the number of reactions, compartmentalized reactions, transport reactions and genes between our models and those in the literature in Figure 2. In the case of the number of reactions, compartmentalized reactions and transport reactions in the Full and Evidenced models for both species, we show that the models created in this work are larger than the published models, with the exception of the model published by Mintz-Oron et al. Our models are larger than other published models primarily due to the more comprehensive database of biochemistry and plant annotations from which we generate our models, as well as the inclusion of recent database updates in our new model. The model published by Mintz-Oron et al. is larger still generally because it was expanded to include many computationally predicted compartmentalized reactions and transporters.

#### Compartments

**Figure 3:**
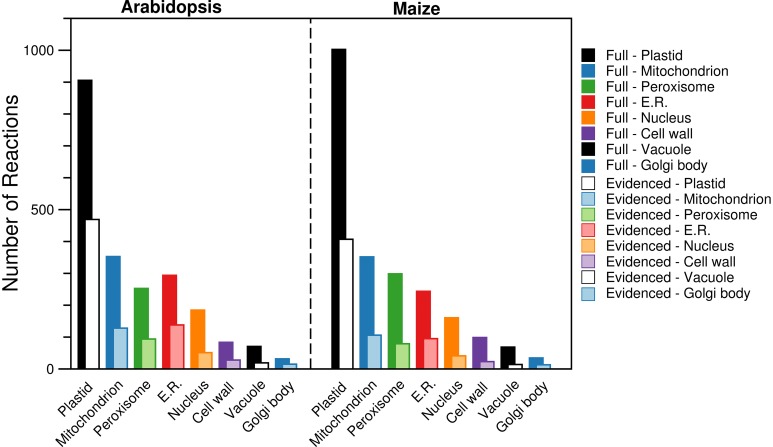
**Comparison of the number of reactions found in each of the compartments in the Full and Evidenced metabolic reconstructions for *Arabidopsis* and maize**. Evidence from AraCyc, PPDB, and SUBA was used to assign each reaction to different compartments. The substantial difference in the number of reactions in each compartment for the Full and Evidenced models is a result of the large number of gene-reaction associations in the Full model, which in turn is a result of the many computational predictions used to make the associations. As such, the use of protein localization evidence to assign reactions to compartments is far more reliable with the use of evidence for the gene-reaction associations in the Evidenced model. ER, Endoplasmic reticulum.

**Figure 4:**
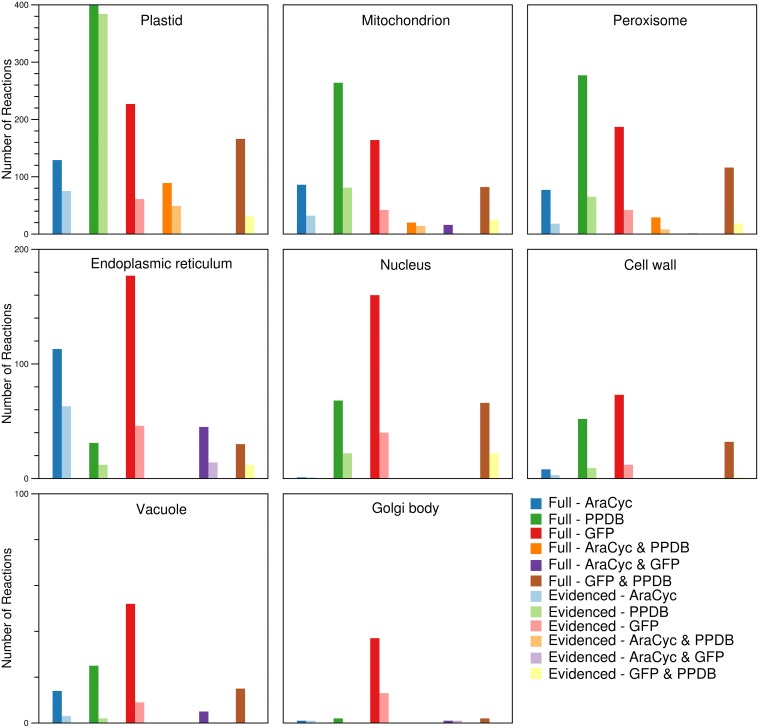
**Comparison of the sources responsible for the number of reactions found in each of the compartments in the Full and Evidenced metabolic reconstructions for maize**. The assignment of reactions to compartments is done by using evidence from AraCyc, PPDB, and SUBA. In general, the number of reactions in different compartments appears to be mostly influenced by a single source. More reactions are assigned by AraCyc to the endoplasmic reticulum; by PPDB to the plastid, mitochondrion, and peroxisome; and by GFP experiments listed in SUBA to nucleus, cell wall, vacuole, and Golgi body. The pairing of PPDB and SUBA shares evidence more frequently in all compartments with the exception of the endoplasmic reticulum where there is more agreement between AraCyc and SUBA.

By using the protein localization data collected from various sources, we were able to confirm the presence of ~2000 reactions in eight compartments (plastid, mitochondrion, peroxisome, endoplasmic reticulum, nucleus, cell wall, vacuole, and Golgi body). We collected gene localization data for 12,398 *Arabidopsis* genes and 8737 maize genes for eight compartments in the metabolic reconstructions (see Materials and Methods), and we added reactions to the appropriate compartment whenever they were associated with a localized gene. We find that the gene localization data led to more than 700 reactions being placed in new locations that are not otherwise designated in the databases and published models used as sources; the “None” column in Figure 1 indicates this. In the next Section, we highlight two reactions as an example of this. We show a breakdown of the number of reactions found in each compartment (Figure 3), and this highlights that the majority of the reactions are found in the plastid. Furthermore, we qualitatively examined the contribution of each database to the localization of reactions (Figure 4). The total number of reactions assigned to any compartment in the Full maize metabolic reconstruction by PPDB data is 1675, by GFP data is 1077, and by AraCyc data is 429. The PPDB data accounts for more reactions in the plastid, mitochondrion, and peroxisome, and the GFP data accounts for more reactions in the remaining compartments. Whilst there is some agreement between the sources, the number of reactions assigned to a compartment by PPDB or GFP alone is a validation of our decision to use multiple sources of evidence-based localization data.

#### Evidence for Gene-Reaction Associations

As stated above, we wish to refine our Full metabolic reconstructions to only contain reactions with reliable evidence for gene-reaction associations. Almost every gene-reaction association found in KEGG, and in any plant BioCyc databases that is not AraCyc, are computationally predicted (Zhang et al., 2010; Nakaya et al., 2013; Kanehisa et al., 2014; Seaver et al., 2014). Additionally, in many of the cases, and this problem is particularly acute in plants, the set of computationally predicted genes associated with reactions may be homologous, but do not perform the same catalytic function (i.e., they are out-paralogs). The large number of gene-reaction associations in the Full metabolic reconstruction for maize highlights this problem because maize, as a species, had a recent whole-genome duplication leading to additional paralogs (Schnable et al., 2009). It is important to identify the correct gene-reaction associations, because the genes duplicated by whole-genome duplication in maize appear to be down-regulated (Schnable and Freeling, 2011; Schnable et al., 2011).

We tackled this problem of over-annotation in two steps. First we included the gene-reaction associations for which there is evidence from two primary sources, AraCyc and PlantSEED (Mueller et al., 2003; Zhang et al., 2010; Seaver et al., 2014). The PathwayTools software enables users to assign evidence codes for gene-reaction associations, and in particular we were able to weed out all the gene-reaction associations where the evidence codes indicated that only a computational prediction was made. The PlantSEED project manually reviewed many of the gene-reaction associations found in AraCyc and elsewhere (Seaver et al., 2014), but also included many carefully reviewed in-paralogs (Sonnhammer and Koonin, 2002; Seaver et al., 2014), thus allowing us to include a greater number of gene-reaction associations in our metabolic reconstructions. By using these sources of evidence, we produced an evidence-based metabolic reconstruction for *Arabidopsis* that contained only those reactions for which there was gene-reaction association evidence from AraCyc and PlantSEED, which we denote as “Evidenced.” The Evidenced metabolic reconstruction for *Arabidopsis* is smaller, with 2801 reactions, and a smaller number of gene-reaction associations (Table 1). The number of reactions in the Evidenced metabolic reconstruction is 44% that of the Full metabolic reconstruction, but the number of gene-reaction associations is 26%, which is an indication of how many computational predictions are made for genes associated with reactions which otherwise have evidence for their associations with other genes.

In the second step of our model refinement, we considered the lack of evidence for any other species, given that much biochemical research in plants has been on *Arabidopsis* as a model organism. As a result, there exist only a tiny number of gene-reaction associations with evidence in MaizeCyc and CornCyc combined, and to create an Evidenced model for maize, one must consider propagating the gene-reaction associations from *Arabidopsis*. In order to avoid the pitfall of over-annotation, and yet create a reliable set of gene-reaction associations for maize, we used the same very conservative approach we applied to EnsemblCompara protein families in the PlantSEED project, described below (Vilella et al., 2009; Seaver et al., 2014). This approach greatly reduced the number of maize orthologs found in the same protein family as the *Arabidopsis* genes found in the Evidenced *Arabidopsis* metabolic reconstruction. In doing so, we are able to create an Evidenced metabolic reconstruction for maize by adding to the model only the reactions for which the associated genes have orthologs in the Evidenced metabolic reconstruction for *Arabidopsis*. The Evidenced metabolic reconstruction for maize has 2631 reactions and, ~30,000 fewer gene-reaction associations than found in the Full metabolic reconstruction (~84%; Table 1).

We highlight the utility of our approach with an example involving two reactions from the mevalonate pathway. Simkin et al. report, using YFP-fused constructs, that Phosphomevalonate kinase (PMK) and Mevalonate diphosphate decarboxylase (MVD) localize to the peroxisomes (Simkin et al., 2011). The complementary reactions for these two enzymes are found in AraCyc, MaizeCyc, and CornCyc, albeit without any localization data attached, and with experimental evidence only available for one enzyme in AraCyc. Thus, only one reaction (MVD) would be included in the Arabidopsis model and would only be cytosolic. The evidence for the gene-reaction associations is found in PlantSEED in the form of manual curation, and leads to both reactions being included in the Arabidopsis model. The results for the enzyme localization from Simkin et al. are found in SUBA, and the two reactions were therefore correctly added to the peroxisome in the Arabidopsis model. Finally, the use of EnsemblCompara protein families as described above leads to the correct maize genes being associated with the same reactions, and the reactions being thus added to the peroxisome in the maize model.

We generated a corresponding metabolic model for all four of our metabolic reconstructions by adding a biomass equation matching that used by the PlantSEED and containing more than 90 compounds. We also utilized a new pathway gapfilling method (see Materials and Methods) that attempts to generate biomass and simultaneously activate all reactions with associated genes. The pathway gapfilling recommended reactions to add to our models to produce biomass and improve the function of all the pathways included in the model. We tested our gapfilled models by simulating growth on heterotrophic media in the KBase environment before applying the transcript profiles.

### Transcriptome-based metabolic reconstructions of maize

#### Maize Transcriptomics

**Figure 5:**
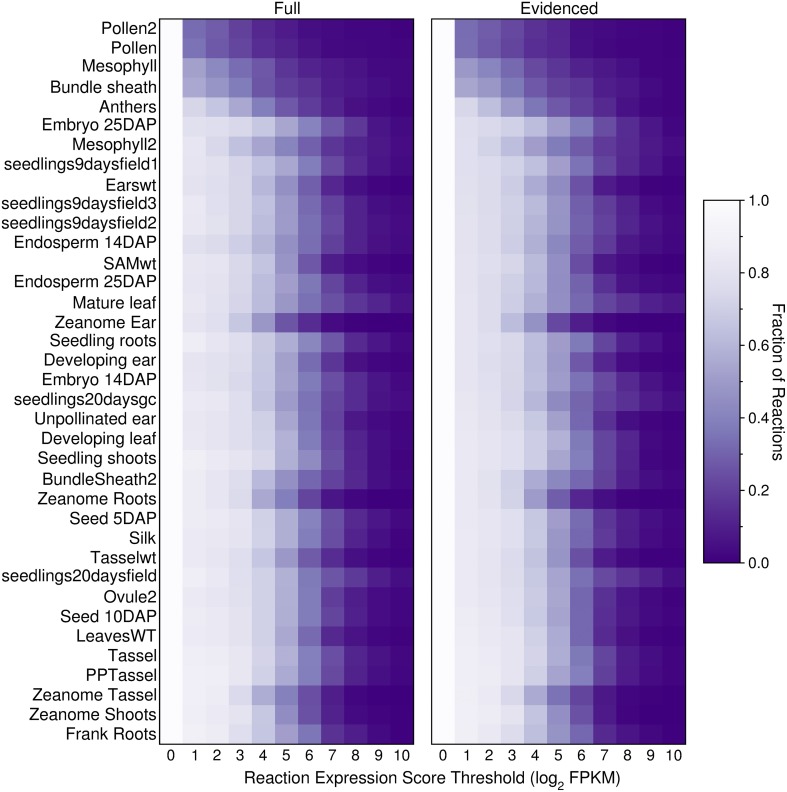
**Fraction of active reactions in the Full and Evidenced metabolic reconstructions for maize at different thresholds**. An expression score is computed for each reaction (Equations 7–9) using maize transcript profiles from qTeller (http://qteller.com). The transcript profiles are ordered by the sizes of the resulting metabolic reconstruction when the threshold applied is one, thus the smaller reconstructions with fewer active reactions are positioned at the top of the figure. Although the Evidenced metabolic reconstruction has half the number of reactions found in the Full metabolic reconstruction, both models appear to shrink at similar rates when increasing the threshold. Two sets of tissues, in general, have more inactive reactions at lower thresholds: (1) reproductive tissues, such as pollen and anthers, as well as tissues consisting of single cell types such as mesophyll and bundle sheath, and (2) tissues which originated from the Zeanome project.

We built transcriptome-based metabolic reconstructions of maize, derived directly from the gapfilled genome-scale Evidenced metabolic model, such that each transcriptome-based model will be a subset of the Evidenced metabolic model. To generate these transcriptome-based metabolic reconstructions, we used RNA-Seq data collated at qTeller (http://qteller.com/, downloaded on 02/04/2014). The data consists of 37 experiments from nine sources, covering a range of cells, tissues, organs, and conditions. As an initial exploration of how the transcript profiles may affect a transcriptome-based model, we computed, for each of the datasets, and at 10 different thresholds, the number of reactions in the genome-scale Full and Evidenced metabolic models for maize that would be active in the organ or tissue, and conditions from which the transcript profiles were retrieved (Figure 5). The threshold was applied to the reaction expression scores (Equations 7–9), and as the threshold increases, the number of reactions that would be active in the resulting metabolic reconstruction decreases. The results show that the smallest metabolic reconstructions are derived either from data from specific cell types (mesophyll and bundle sheath) or highly reproductive tissues (pollen and anthers); the other tissues and organs with larger reconstructions encompassed multiple cell types and in general, up to a threshold of four, show little difference in the sizes of the resulting metabolic network. Furthermore, qualitatively, it appears that the relative change in the network sizes is similar across organs and tissues in both the Full and Evidenced metabolic models. Finally, using several of the transcript profiles from the same source appears to consistently result in metabolic networks that are relatively smaller, notably those from the Zeanome dataset (http://www.ncbi.nlm.nih.gov/Traces/sra/?study=SRP011480), which is an important reminder that, when performing *in silico* experiments using transcript profiles, one must ensure they come from the same source.

**Figure 6:**
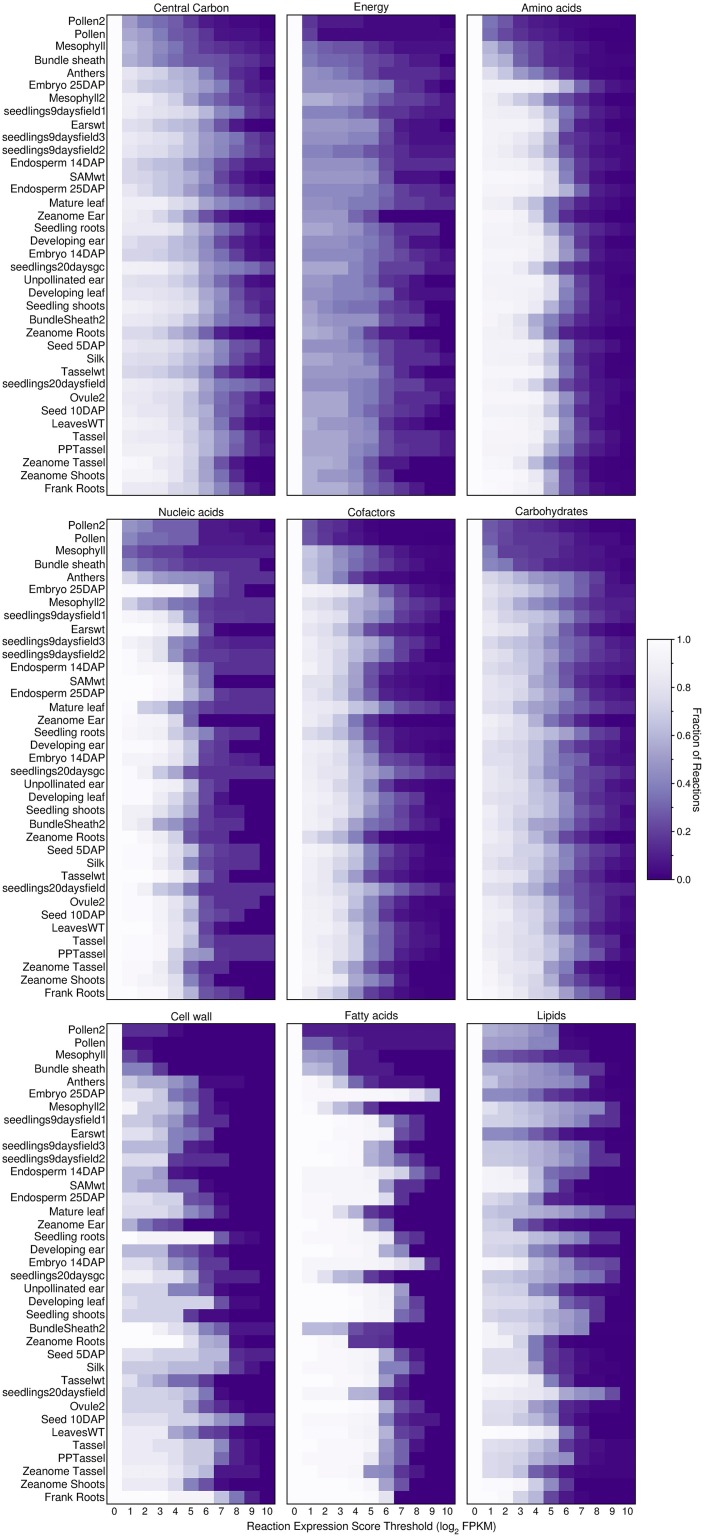
**Fraction of active reactions involved in different categories of plant primary metabolism at different thresholds**. An expression score is computed for each reaction (Equations 7–9) using maize transcript profiles from qTeller (http://qteller.com). The results shown here are for the Evidenced metabolic reconstruction for maize. The figure indicates that between categories of primary metabolism and from tissue to tissue, the fraction of active reactions exhibits substantial variation. Some tissues have a high fraction of reactions active at a high threshold within certain categories, for example, within the tissue sample named “Embryo_25DAP” (25 days after pollination) and within the category of Fatty acids. This result reflects a known biological function of the embryo, as a store of lipids. The high degree of variation in the number of active reactions at different thresholds in plant primary metabolism is a strong indication that using a single gene expression threshold across an entire metabolic reconstruction may produce undesired results.

**Figure 7:**
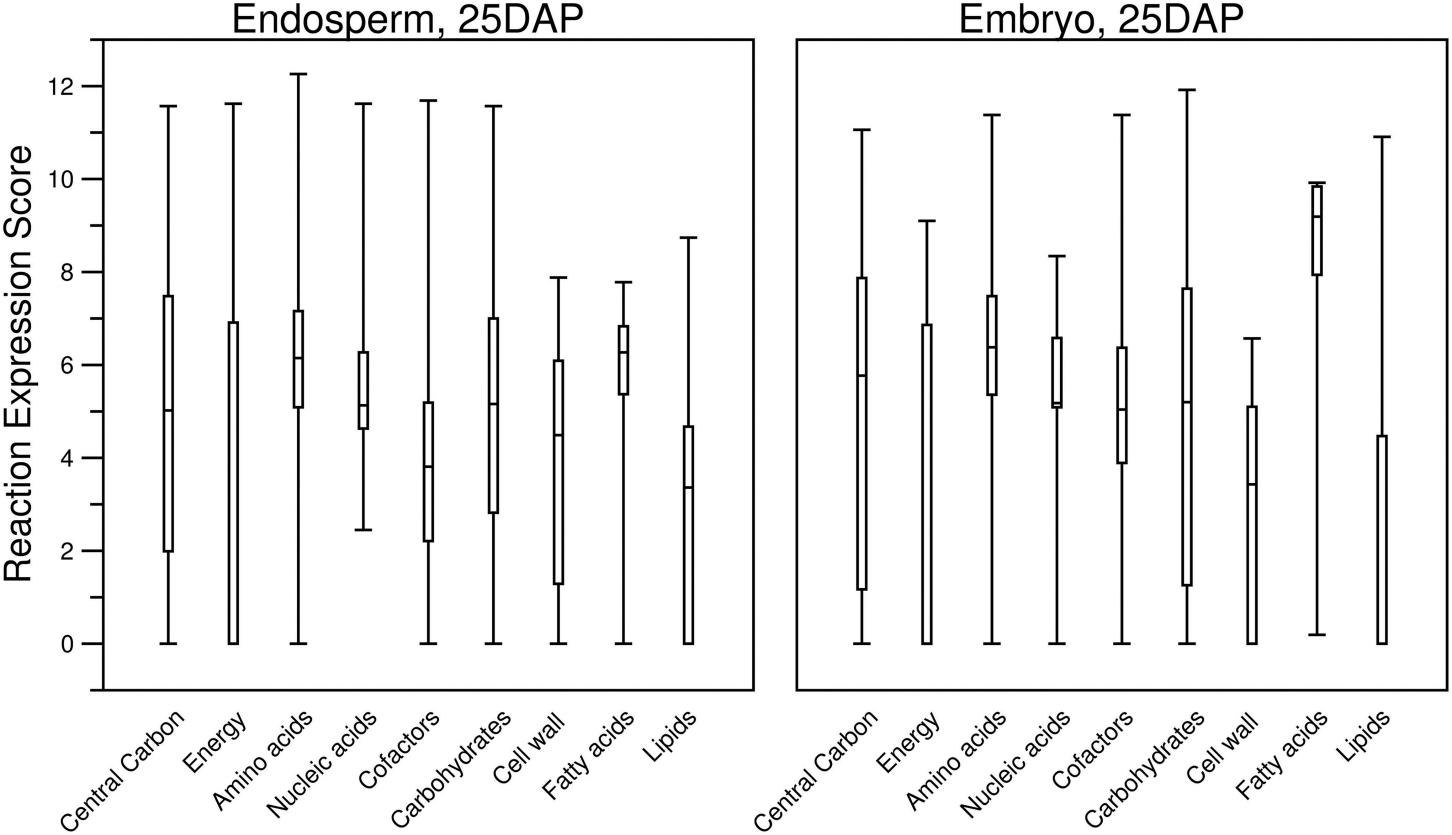
**Boxplots describing the distribution of computed reaction expression scores (Equations 7–9) from the transcript profiles of two tissues and within the different categories of plant primary metabolism**. Almost every category contained at least one reaction with a reaction expression score of zero. Furthermore, for the “Energy” and “Lipids” categories, more than half of the reaction expression scores are zero. It can be seen that the median reaction expression scores for “Embryo, 25DAP” are higher, which supports the observation made for this tissue in the previous figure (Figure 6). Additionally, the lower quartiles of the reaction expression scores in the “Carbohydrates” and “Cell wall” categories are higher for “Endosperm, 25DAP.” Both of these categories include pathways involved in sugar metabolism, and this supports the known biological function of the endosperm as a storage of starch.

To investigate further, we explored how the threshold creates gaps in the primary metabolism of transcriptome-based models. We aggregated the various pathways under nine different categories of primary metabolism as defined by the PlantSEED project (Seaver et al., 2014) and we explored how these pathways shrink in size as the threshold is increased (Figures 6, 7). Overall, within each pathway category, a similar pattern is observed where the sex organs and single-cell transcript profiles result in the smaller metabolic model, and for all transcript profiles, there appears to be a similar decrease in the sizes of the pathways. However, it is notable that this pattern varies from category to category and from organ to organ or tissue to tissue. Many essential reactions that may be necessary for a derived metabolic model to operate may be inactivated by the use of a simple expression threshold. For instance, within almost every category, there are reactions for which the computed expression score is zero or constitutively low (Figure 7), but the reactions are essential. Reactions in the “Fatty acids” category would appear to be the most impervious to the use of a low threshold as many, if not all, of the reactions appear to exhibit a medium to high expression score across most organs and tissues. A notable example is the set of reaction expression scores computed from the transcriptome labeled Embryo_25DAP (25 days after pollination), which matches our understanding of the embryo typically being rich in lipids. As it is therefore not reasonable to use a simplistic approach to generate transcriptome-based metabolic models, we thus develop a novel method for applying the gene expression levels in transcript profiles directly to the genome-scale metabolic model (see Materials and Methods). The method attempts to activate every reaction that is associated with a highly expressed gene whilst minimizing activity of reactions associated with minimally expressed genes. The results of the generation of these models from transcript profiles using this method are found in Section Generating the Transcriptome-Based Metabolic Models. However, first we address the derivation of new biomass compositions to represent the leaf, endosperm and embryo tissues.

#### High-Quality Maize Biomass Equation for Leaf, Endosperm and Embryo Tissue

One use for the metabolic models we build is to predict the biosynthesis of plant biomass components. This is done by creating a specialized biomass composition reaction that contains each of the biomass components in relative proportions, and by “maximizing” biomass production when simulating growth in the metabolic model. All of the prior published metabolic models for plants have assumed a basic biomass composition that contained mostly primary metabolites. Little emphasis was placed on the diversity of compounds that a plant biosynthesizes. For our transcriptome-based metabolic models, we aim to distinguish between the functions of the models by providing a high-quality biomass composition reaction representing the organ or tissue from which the modeled transcriptomes were collected. We constructed these reaction based on an extensive literature search. Here we describe a biomass that contains more cofactors and fatty acids, supported by almost 30 literature references, including detailed quantifications. The following paragraphs briefly described the biomass composition along with the relevant references.

##### Amino acids

The biomass fraction attributable to protein is estimated to be 8 and 11.6% of dry weight in endosperm and embryo, respectively (Ingle et al., 1965). To quantify the relative contribution of each amino acid in the endosperm, the total amino acid context determined experimentally by Misra et al. (1972) was used with two exceptions. Firstly, the cysteine content was doubled as the reported value concerned cystine. Secondly, the glutamate:glutamine and aspartate:asparagine ratios were deduced from the composition of mature Zein proteins (Wu et al., 2012) to estimate their individual contribution. For composition of amino acids in embryo, the sequences of two globulins were used, which account for 20% of total embryo protein (Belanger and Kriz, 1989; Wallace and Kriz, 1991). Water loss due to formation of the peptide bond was taken into account.

##### Nucleic acids

The biomass fraction attributable to DNA was reported to be 0.038 and 0.015% in endosperm and embryo, respectively, while that attributable to RNA was reported to be 0.3 and 0.1% in endosperm and embryo, respectively (Ingle et al., 1965). The biomass fraction attributed to each nucleotide was estimated using published GC content (Haberer et al., 2005).

##### Carbohydrates

The endosperm biomass fraction attributable to carbohydrates was calculated to be about 90% of dry mass (Ingle et al., 1965; Alonso et al., 2011). Of this carbohydrate fraction, 77.6% is starch, 16.6% is cell walls (Alonso et al., 2011) and the remaining 5.8% is free sucrose, fructose, and glucose (Ingle et al., 1965). The reported composition of endosperm cell walls (Dewitt et al., 1999) was used to calculate the quantities of the majority of the monosaccharides. The embryo biomass fraction attributable to carbohydrates is calculated to be 58.5% (Rolletschek et al., 2005; Alonso et al., 2010). Of this carbohydrate fraction, 49.6% is starch, 42.7% is cell walls (Alonso et al., 2010) and the remaining 7.7% is free sucrose, fructose, and glucose (Rolletschek et al., 2005). The reported composition of cell walls (McCann et al., 2007) was used to calculate the quantities of the majority of the monosaccharides and the ratio of monosaccharides found in the leaf (Penning de Vries et al., 1974) was used to calculate ribose, glucuronate, and galacturonate content.

For both endosperm and embryo, the galactose, glycerol, and sulfoquinovose biomass fraction was estimated using values for galactolipids, glycerolipids, and sulfolipids, respectively (see the Section Lipids and Sterols). Finally, further evidence was used to deduce the biomass fraction of inositol (Teas, 1954).

##### Phenolic compounds

The cell wall of maize is considered to contain two main types of phenolic derivatives: p-coumaric acid and ferulic acid (Assabgui et al., 1993; Saulnier et al., 1995).

##### Vitamins and cofactors

As key components of metabolism, we emphasized biosynthetic pathways of cofactors more than the published metabolic models, and specified the biomass fraction assigned to each of the B vitamins and other cofactors with greater accuracy. The list of vitamins and cofactors included biotin, thiamin diphosphate, NAD and derivatives, FAD and FMN, coenzyme A, 4-phosphopantetheine, tetrahydrofolate and its derivatives, α-tocopherol, ascorbate, ubiquinone-9, lipoic acid, heme, and pyridoxal-5′-phosphate (Cameron and Teas, 1948; Teas, 1954; Giri et al., 1960; Ingle et al., 1965; Metz et al., 1970; Weber, 1987; Battey and Ohlrogge, 1990; Shannon et al., 1996; Szal et al., 2003; Tumaney et al., 2004; Shi et al., 2005; Drozak and Romanowska, 2006; Hu et al., 2006; Naqvi et al., 2009; Perez-Lopez et al., 2010; Richter et al., 2010; Enami et al., 2011; Spielbauer et al., 2013; Seaver et al., 2014).

##### Pigments

Two pigments were included in our endosperm and embryo biomass: β-carotene, and lutein (Weber, 1987).

##### Lipids and sterols

Lipids represent 1.5 and 32.6% of the biomass of endosperm and embryo, respectively (Weber, 1979). The biomass composition of fatty acids and sitosterol, campesterol, stigmasterol, and phytosphingosine in this study were based on those reported by Weber (1979). Galactose, glycerol, and sulfoquinovose content were also calculated based on the lipid composition.

##### Carboxylic acids and other compounds

Many other compounds compose plant biomass, and we included here a list of a subset of these for which a value is reported in the literature: *cis*-aconitate, citrate, malate, oxaloacetate lactate (Skogerson et al., 2010; Rolletschek et al., 2011), and S-adenosylmethionine (Apelbaum and Yang, 1981). Choline and ethanolamine were estimated from the values for phosphatidylcholine and phosphatidylethanolamine, respectively. Finally, the mineral content of the biomass was set at 5%, split evenly between potassium and chloride (Penning de Vries et al., 1974).

#### Generating the Transcriptome-Based Metabolic Models

We used the novel transcriptome-based gapfilling approach (see Materials and Methods) along with three separate transcript profiles to generate metabolic models that are specific to the leaf, endosperm and embryo, which are named “Leaves 20-day old seedling – field,” “Endosperm 25 days after pollination,” and “Embryo 25 days after pollination” (Davidson et al., 2011) http://www.ncbi.nlm.nih.gov/bioproject/80041). We used these three transcript profiles in particular because they came from the same experiments and, therefore, were processed in a similar manner.

We applied the three transcriptome profiles separately to the Evidenced metabolic model of maize (see Materials and methods Section) to generate three separate metabolic models that can grow in heterotrophic media. All three metabolic models contained an average of 2302 reactions (Table 1), which is 88% of the number of reactions in the Evidenced model, and there are 2153 reactions that are found in all three of them. By comparison, the final compartmentalized model created by Mintz-Oron et al. (2012) for *Arabidopsis* has 3508 reactions and the resulting tissue-specific models generated from their work has on average 2848 reactions, which is 81% of the reactions in their full model. This result indicates that our approach, while using a full set of gene expression data for the maize transcript profiles to generate smaller models, results in models whose sizes are similar to other work on generating organ and tissue-specific models for a plant.

### Comparison of fluxes in tissue-specific metabolic reconstructions to fluxomics data

We have described a process that generates and refines metabolic models in three steps, generating metabolic models at each step. We can now show how these metabolic models not only compare with fluxomics data, but how that comparison improves at each step, resulting in transcriptome-based models with the closest fit to the original fluxomics data.

The experimental data we used were fluxes for central carbon metabolism estimated using ^14^C labeling in two different tissues, the embryo and endosperm (Alonso et al., 2010, 2011). The reactions from these two studies were matched to the reactions in the models, and we used the approach described in Section Comparison with Estimated Fluxomics Data for Embryo and Endosperm to fit the fluxes within the models to the experimentally determined fluxes. We report the Spearman correlation and its *p*-value in Table 2, showing that the correlation is high for both transcriptome-based models. This result indicates that the central carbon metabolism of the models generated in this work is able to perform as observed in the original tissues. The reactions used here have a median expression score of 6.60 and 7.17 in the embryo and endosperm transcriptomics dataset, respectively, but the lowest expression score is ~1.2 for both tissues. This last statement in turn exemplifies the importance of our approach, in ensuring that reactions with a low expression score are still included in model generated from a transcript profile if considered to be essential for the metabolic functioning of the organ or tissue.

**Table 2:** **Comparison of prior published model of maize with the models generated by this work using the percentage of blocked reactions and the spearman rank correlation coefficient when using fluxomics data (*p*-value in parentheses)**.

**Type/Tissue**	**Blocked reactions (%)**	**Endosperm**	**Embryo**
iRS1563^*^	53	0.69 (2.3 × 10^−3^)	0.46 (7.5 × 10^−2^)
Full	30	0.99 (9.4 × 10^−51^)	0.99 (7.1 × 10^−54^)
Evidenced	21	0.99 (4.7 × 10^−34^)	0.83 (1.7 × 10^−10^)
Evidenced/Endosperm	16	0.99 (1.3 × 10^−29^)	n/a
Evidenced/Embryo	16	n/a	0.83 (8.6 × 10^−10^)

* Saha et al. (2011).

## Discussion

In this manuscript, we created a total of seven metabolic reconstructions for two species (see Supplementary Material). In succession, we created two Full metabolic reconstructions for *Arabidopsis* and maize, comprised of many possible sources of plant biochemistry reconciled into single large networks. These Full models also included many predicted gene-reaction associations, a subset for which we found evidence either in the literature or via human inference, and we used these to create a more reliable metabolic reconstruction for the two species. Finally, via use of a novel, simple and fast organ and tissue-specific pathway gapfilling method, along with well-curated biomass for the leaf, endosperm and embryo, we generated three metabolic models specific for these organ and tissues. The evidence that we used, for both the genes whose products catalyze the reactions and the localization of gene products in different compartments, is comprehensive and reliable.

Our approach allows us to create relatively large metabolic reconstructions that compare favorably to the prior published metabolic models, albeit with a smaller set of gene-reaction associations. This enables us to apply transcriptome data with a high degree of confidence. The approach is validated by the fact that the embryo and endosperm models retained nearly every reaction of central carbon metabolism. This was done both by the body of evidence available for the gene-reaction associations, and the pathway gapfilling method which included reactions with a low expression score, but were essential to the models. Finally, it was shown that the same models can be active and able to replicate the activity observed in published experimental fluxomics datasets. To date, we believe we are the first to apply such wide-ranging body of evidence to the generation of large-scale metabolic reconstructions.

All of our work was carried out through the DOE Systems Biology Knowledgebase (KBase; http://kbase.us/), an open software and data platform that aim to enable researchers to predict and ultimately design biological function. The data is publicly available within KBase workspaces named “Maize_Tissue_Models” (https://narrative.kbase.us/functional-site/#/ws/objects/Maize_Tissue_Models) and also via the PlantSEED website (http://plantseed.theseed.org). The KBase software environment allows researchers to copy the individual metabolic models and to explore the models using the suite of modeling tools available.

### Conflict of interest statement

The authors declare that the research was conducted in the absence of any commercial or financial relationships that could be construed as a potential conflict of interest.

## Supplementary material

### Data Sheet1.ZIP

Zip file of metabolic models in SBML format. All seven metabolic models, the Full metabolic and Evidenced metabolic models for *Arabidopsis* and maize and the three tissue-specific metabolic models for maize leaf, endosperm, and embryo are available for download in SBML format.

### Data Sheet2.XLSX

Spreadsheet of metabolic models. An Excel spreadsheet containing details for the seven metabolic models and also containing details of their relationships with prior published models, and the reaction-gene and gene-compartment evidence.
